# Unraveling the Role of *Fusobacterium nucleatum* in Colorectal Cancer: Molecular Mechanisms and Pathogenic Insights

**DOI:** 10.3390/cancers17030368

**Published:** 2025-01-23

**Authors:** Linda Galasso, Fabrizio Termite, Irene Mignini, Giorgio Esposto, Raffaele Borriello, Federica Vitale, Alberto Nicoletti, Mattia Paratore, Maria Elena Ainora, Antonio Gasbarrini, Maria Assunta Zocco

**Affiliations:** 1 Internal Medicine, Fondazione Policlinico Universitario “A.Gemelli” IRCCS, Università Cattolica del Sacro Cuore, 20123 Rome, Italy; linda.galasso@guest.policlinicogemelli.it (L.G.); fabrizio.termite@libero.it (F.T.); irene.mignini@guest.policlinicogemelli.it (I.M.); giorgio.esposto@guest.policlinicogemelli.it (G.E.); raffaeleborr@gmail.com (R.B.); federica.vitale@guest.policlinicogemelli.it (F.V.); alberto.nicoletti@policlinicogemelli.it (A.N.); mattia.paratore@guest.policlinicogemelli.it (M.P.); mariaelena.ainora@policlinicogemelli.it (M.E.A.); antonio.gasbarrini@unicatt.it (A.G.); 2 CEMAD Digestive Disease Center, Fondazione Policlinico Universitario “A.Gemelli” IRCCS, Università Cattolica del Sacro Cuore, 20123 Rome, Italy

**Keywords:** *F. nucleatum*, CRC, IL-1β, IL-6, TNF-α, FadA, EMT

## Abstract

*Fusobacterium nucleatum*, a gram-negative anaerobic bacterium, has emerged as a significant player in colorectal cancer (CRC) pathogenesis. The bacterium causes a persistent inflammatory reaction in the colorectal mucosa by stimulating the release of pro-inflammatory cytokines like IL-1β, IL-6, and TNF-α, creating an environment conducive to cancer progression. *F. nucleatum* binds to and penetrates epithelial cells through adhesins such as FadA, impairing cell junctions and encouraging epithelial-to-mesenchymal transition (EMT), which is associated with cancer advancement. Additionally, the bacterium modulates the host immune system, suppressing immune cell activity and creating conditions favorable for tumor growth. Its interactions with the gut microbiome contribute to dysbiosis, further influencing carcinogenic pathways. Evidence indicates that *F. nucleatum* can inflict DNA damage either directly via reactive oxygen species or indirectly by creating a pro-inflammatory environment. Additionally, it triggers oncogenic pathways, especially the Wnt/β-catenin signaling pathway, which promotes tumor cell growth and longevity. Moreover, *F. nucleatum* alters the tumor microenvironment, impacting cancer cell behavior, metastasis, and therapeutic responses. The purpose of this review is to elucidate the molecular mechanisms by which *F. nucleatum* contributes to CRC. Understanding these mechanisms is crucial for the development of targeted therapies and diagnostic strategies for CRC associated with *F. nucleatum*.

## Simple Summary

*Fusobacterium nucleatum*, a gram-negative anaerobic bacterium, plays a pivotal role in colorectal cancer (CRC) pathogenesis. It induces chronic inflammation via cytokines such as IL-1β, IL-6, and TNF-α, fostering tumor progression. Through adhesins like FadA, *F. nucleatum* disrupts cell junctions and promotes epithelial-to-mesenchymal transition (EMT). The bacterium suppresses immune responses, exacerbates gut dysbiosis, and activates oncogenic pathways, notably Wnt/β-catenin signaling. It also inflicts DNA damage directly through reactive oxygen species or indirectly via inflammation. By altering the tumor microenvironment, *F. nucleatum* impacts metastasis and therapy outcomes. Understanding these mechanisms is essential for advancing CRC therapies and diagnostics.

## 1. Introduction

*Fusobacterium nucleatum* (*F. nucleatum*), a Gram-negative, obligate anaerobic bacterium, has garnered significant attention for its pivotal role in the development and progression of colorectal cancer (CRC) [1]. Initially recognized as a common resident of the human oral cavity, *F. nucleatum* has since been implicated in various pathological processes associated with CRC, including tumorigenesis, metastasis, and resistance to therapy [2,3]. Its pro-carcinogenic effects are primarily linked to its interactions with host cells and its ability to modulate the immune microenvironment, positioning it as a promising biomarker and therapeutic target for CRC [1,4].

The accumulation of *F. nucleatum* has been linked to the advancement, growth, and unfavorable outcomes of CRC. Globally, CRC is the third most common malignancy and ranks fourth among causes of cancer-related mortality. Projections indicate a 60% increase in CRC diagnoses by 2030 [5,6,7]. Recent studies suggest a significant connection between gut microbial imbalances and the development of CRC [8,9,10]. In particular, *F. nucleatum* has been found to be more prevalent in CRC tissues compared to normal tissues nearby [2,11]. Additionally, molecular features like the CpG island methylator phenotype, microsatellite instability, and a lower density of CD3+ T-cells have been linked to increased levels of *F. nucleatum* in CRC samples [4,12].

Surgical intervention is the primary treatment for early stage CRC, while adjuvant options like chemotherapy and targeted therapies are essential for advanced stages. Nevertheless, resistance to chemotherapy remains a major obstacle, driven by factors such as genetic mutations, epigenetic alterations, and changes in the tumor microenvironment [13,14,15]. Recent findings suggest that *F. nucleatum* contributes to chemoresistance by modifying the tumor microenvironment and regulating the expression of genes critical to drug response [4,16].

The literature presents different data, as some studies have indicated that elevated levels of *F. nucleatum* correlate with reduced survival in over 1000 CRC patients, while Oh et al. highlighted the prognostic role of *F. nucleatum* in individuals undergoing adjuvant chemotherapy [17,18]. These discrepancies underscore the necessity for in-depth research into the associations between *F. nucleatum* and various CRC subtypes.

This review aims to provide a comprehensive analysis of the molecular mechanisms through which *F. nucleatum* contributes to CRC. A deeper understanding of these mechanisms is essential for developing targeted therapeutic and diagnostic strategies to address CRC linked to *F. nucleatum.*

## 2. The Pro-Tumorigenic Role of *Fusobacterium nucleatum* in Colorectal Cancer: Mechanisms of Adhesion, Signaling, and Epigenetic Alteration

The pro-tumorigenic effects of *F. nucleatum* are primarily mediated by its adhesins, FadA and Fap2, which enable the bacterium to adhere to and invade human epithelial and endothelial cells [19,20].

The FadA protein binds to E-cadherin in epithelial cells and VE-cadherin in endothelial cells, two key cell-junction molecules [19]. FadA’s interaction with VE-cadherin disrupts endothelial cell-cell junctions, increasing vascular permeability and facilitating the hematogenous spread of *F. nucleatum* to distant sites. In parallel, Fap2 acts as an autotransporter, promoting bacterial adhesion through recognition of overexpressed Gal-GalNAc on tumor epithelial cells [21,22].

Through these molecular interactions, *F. nucleatum* triggers intracellular signaling cascades that promote cancer cell survival and proliferation.

A key pathway influenced by FadA is the WNT/β-catenin signaling pathway, which governs cell growth, differentiation, and survival. Activation of this pathway by *F. nucleatum* has been associated with poor outcomes in CRC patients due to its role in driving uncontrolled tumor growth and metastasis [23,24]. Moreover, *F. nucleatum*, through the disruption of the E-cadherin/β-catenin complex, drives epithelial cells toward a mesenchymal-like phenotype, thereby enhancing their invasive potential [4].

Additionally, *F. nucleatum* modulates Annexin A1, a regulator of the WNT/β-catenin pathway and a biomarker of poor prognosis in cancer. The interaction between FadA and Annexin A1 establishes a positive feedback loop, amplifying tumorigenic signaling and further contributing to CRC progression [25,26,27].

Recent studies have revealed the presence of a protein known as RadD, which enables *F. nucleatum* to influence CRC cells through a targeted interaction with the CD147 receptor [28,29]. This interaction notably stimulates the PI3K-AKT-NF-κB-MMP9 signaling pathway, resulting in the release of matrix metalloproteinases (MMPs) that are essential for CRC cell proliferation, migration, and invasion [30,31].

*F. nucleatum* contributes to the progression of CRC through other mechanisms involving epigenetic changes, DNA repair interference, and inflammation [12]. It impedes DNA repair by inhibiting NEIL2 glycosylase and disrupting the Chk2 signaling pathway, which results in DNA double-strand breaks (DSBs) and defects in repair pathways like non-homologous end joining (NHEJ) [32]. These alterations lead to microsatellite instability (MSI), including MSI-L or elevated microsatellite alterations at selected tetranucleotide repeats (EMAST), and facilitate the relocation of the mismatch repair protein MSH3 [14,15,16,17,18,19,20,21,22,23,24,25,26,27,28,29,30,31,32,33]. *F. nucleatum* is linked to the CpG island methylator phenotype (CIMP) and the hypermethylation of tumor suppressor genes (TSGs), a process driven by enhanced DNA methyltransferase activity [4,14]. This epigenetic alteration typically leads to microsatellite instability (MSI-H) and the silencing of mismatch repair genes, such as MLH1, often in conjunction with BRAF mutations [33,34].

Moreover, *F. nucleatum* exacerbates inflammation by inducing the production of reactive oxygen species (ROS) and inflammatory cytokines, further altering DNA methylation patterns and causing DNA damage [35,36]. While MSI-H CRCs are generally linked to better clinical outcomes due to immune activation, high levels of *F. nucleatum* are associated with poorer prognosis by intensifying inflammation [4,37]. Overall, *F. nucleatum*’s role in promoting CIMP-positive CRC and its impact on DNA repair, MSI, and inflammation underline its contribution to CRC progression and aggressiveness [38].

**Figure 1:**
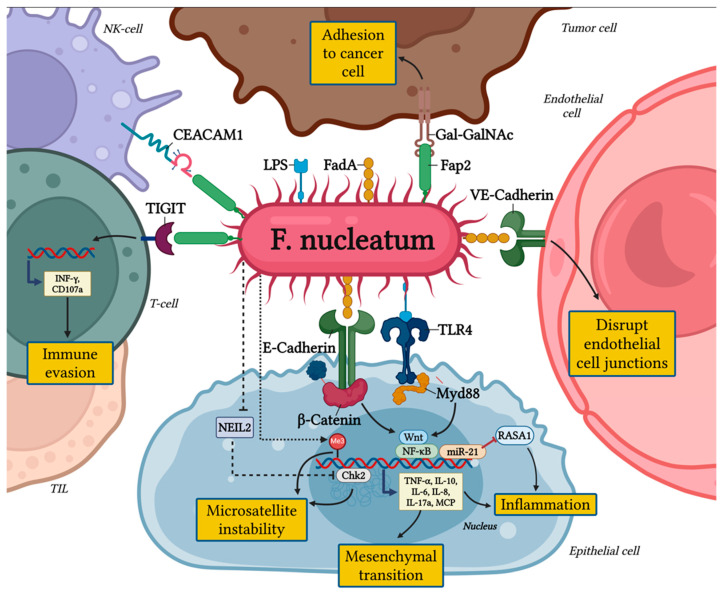
The pro-tumorigenic role of *Fusobacterium nucleatum* in colorectal cancer.

These findings are depicted in Figure 1 and highlight *F. nucleatum* as a crucial risk factor for CRC progression and metastasis, as well as a potential biomarker for poor prognosis.

## 3. Impact of *Fusobacterium nucleatum* on the Tumor Microenvironment

*F. nucleatum* not only exerts direct effects on colorectal cancer (CRC) cells but also profoundly shapes the immune microenvironment by modulating immune responses.

### 3.1. Disruption of Cellular Adhesion and Inflammatory Pathways

As previously discussed, *F. nucleatum* affects epithelial cell adhesion through its adhesin FadA, which interferes with E-cadherin, a crucial protein for maintaining cell-cell connections. This interference leads to the accumulation of β-catenin and activates β-catenin-dependent transcription (CRT), promoting pro-oncogenic pathways such as Wnt signaling and NF-κB. The activation of these pathways results in the production of pro-inflammatory cytokines, including IL-8, IL-10, IL-6, TNF-α, MCP-1, and IL-17A, which create a chronic inflammatory environment [39,40]. This sustained inflammation in turn promotes tumor cell proliferation, survival, and invasion, facilitating the progression of colorectal cancer (CRC) [39]. *F. nucleatum* also upregulates NF-κB activity via miR-21, a microRNA that activates Toll-like receptor 4 (TLR4) and interacts with myeloid differentiation factor 88 (MyD88) [4,41,42]. This cascade suppresses RASA1, a RAS GTPase activator, leading to the accumulation of inflammatory mediators that further stimulate tumor cell proliferation. Experimental data suggest that miR-21 is a critical player in *F. nucleatum*-mediated tumor-promoting inflammation and a potential biomarker for poor CRC outcomes [41,43].

### 3.2. Immune Evasion via TIGIT and CEACAM1

*F. nucleatum* uses the inhibitory receptor TIGIT, which is expressed on T cells, NK cells, and tumor-infiltrating lymphocytes (TILs), to evade immune detection [43]. TIGIT competes with the activating receptor CD226 for binding to CD155, which results in a reduction of NK cell cytotoxicity and T cell activation, thus hindering effective anti-tumor immune responses [44]. Moreover, TIGIT modulates dendritic cells (DCs) by promoting an immunosuppressive environment, increasing IL-10 production while decreasing IL-12 levels [45]. The increased expression of TIGIT also enhances the suppressive function of regulatory T cells (Tregs), further impairing anti-tumor immunity [46]. Clinical evidence links high TIGIT levels with advanced CRC stages, early recurrence, and poorer survival [46,47]. *F. nucleatum*’s Fap2 protein amplifies immune evasion by binding to TIGIT, reducing NK cell cytotoxicity and inducing T cell death [43]. Concurrently, *F. nucleatum* activates CEACAM1, another inhibitory receptor on NK and T cells [43,48]. CEACAM1 activation contributes to T cell exhaustion, marked by diminished levels of IFN-γ and CD107a, key molecules for antitumor activity [49]. These pathways are critical in *F. nucleatum*-mediated immune suppression and tumor progression.

### 3.3. Recruitment and Modulation of Immunosuppressive Cells

*F. nucleatum* selectively recruits myeloid-derived suppressor cells (MDSCs) to the TME [1,50]. MDSCs inhibit T cell proliferation and induce apoptosis through high levels of inducible nitric oxide synthase (iNOS) and arginase-1 [51,52]. Tumor-associated macrophages (TAMs), neutrophils, and regulatory DCs, also recruited by *F. nucleatum*, further promote inflammation, angiogenesis, invasion, and metastasis [53,54]. *F. nucleatum* drives tumor-associated neutrophils (TANs) toward a pro-tumor N2 phenotype via TGF-β signaling. N2 TANs exacerbate tumorigenesis by producing reactive oxygen species (ROS), which cause DNA damage and enhance tumor progression [55,56]. Macrophages influenced by *F. nucleatum* via TLR4 signaling shift toward an M2-like phenotype, which aids tumor progression by dampening adaptive immune responses, encouraging angiogenesis, and facilitating tissue remodeling [57,58]. *F. nucleatum* reduces the density of CD4+ T cells in tumors compared to normal tissues, highlighting its role in suppressing T helper cell-mediated immune responses. This suppression weakens the adaptive immune response, further enabling tumor progression [59,60] (Table 1).

## 4. The Role of *F. nucleatum* in Gut Dysbiosis and Colon Carcinogensis

**Figure 2:**
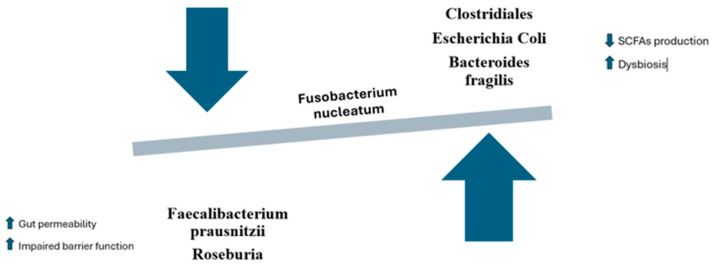
Dysbiosis and modulation of the gut microbiota caused by *Fusobacterium nucleatum*.

The role of *Fusobacterium nucleatum* in intestinal dysbiosis and colon carcinogenesis is a complex phenomenon, involving molecular mechanisms that modulate gut microbial composition, inflammation, and metabolite production [61]. *F. nucleatum* has been linked to reduced bacterial diversity, disrupting the balance between beneficial bacteria, such as *Lactobacillus* and *Bifidobacterium*, and harmful or pro-inflammatory bacteria like Bacteroides fragilis and *Enterococcus* (Figure 2) [62,63]. In dysbiotic conditions, *F. nucleatum* disrupts the activity of beneficial microbes responsible for generating short-chain fatty acids (SCFAs), including acetate, propionate, and butyrate, which are critical for maintaining gut health [64,65,66]. Butyrate, in particular, is essential for gut barrier integrity and energy supply to colonocytes. Its reduction, due to the suppression of key butyrate-producing bacteria like *Faecalibacterium prausnitzii* and *Roseburia*, leads to a weakened intestinal epithelium and increased gut permeability [67,68]. This impaired barrier function facilitates inflammation and further microbial imbalance. *F. nucleatum* also promotes the growth of other harmful microbes, such as pathogenic strains of *Clostridia* and *Escherichia coli*, which are less efficient in producing SCFAs and exacerbate dysbiosis [69,70,71,72]. As a result, SCFA production decreases, contributing to gut dysfunction and compromised immune regulation.

*F. nucleatum* alters SCFA production through competition for substrates and the production of pro-inflammatory metabolites. The fermentation of complex carbohydrates like fiber by beneficial bacteria can be hindered by *F. nucleatum*, which preferentially ferments other molecules, reducing substrate availability for beneficial bacteria [65,73]. Moreover, *F. nucleatum* can promote the production of toxic metabolites such as sulfur compounds and amines from protein fermentation, which can damage the intestinal mucosa and induce an inflammatory state that supports carcinogenesis [71,74].

This reduction in SCFA production, along with the accumulation of toxic and inflammatory metabolites, directly increases the risk of colon cancer, as chronic inflammation and tissue damage are key drivers of cancer development [75].

*F. nucleatum* can also interact with other bacterial species, encouraging the growth of pathogenic strains like *Bacteroides fragilis*, which is linked to an increased risk of colon cancer [76,77]. These interactions occur through several mechanisms, including the synergy of inflammation. *B. fragilis* produces a toxin that triggers the secretion of inflammatory cytokines, further promoting dysbiosis. The co-presence of *F. nucleatum* and *B. fragilis* in the gut creates an inflammatory environment that facilitates tumor progression [78]. Additionally, some strains of *B. fragilis* produce the *B. fragilis* toxin (BFT), which can damage the DNA of colon epithelial cells, inducing mutations that contribute to carcinogenesis [79,80]. This interplay between *F. nucleatum* and other gut bacteria plays a pivotal role in modulating the intestinal environment in ways that promote the development of colon cancer [9].

## 5. Molecular Mechanisms of *Fusibacterium nucleatum* in Chemoresistance and Colorectal Cancer Progression

The effects of *Fusobacterium nucleatum* on the tumor microenvironment and its direct role in tumorigenesis have a significant impact on current colorectal cancer therapies [1,4]. As is well known, the microbiota plays a crucial role in treatment response, and there is ongoing research focused on studying the effects of *Fusobacterium nucleatum* abundance in this context [81]. *Fusobacterium nucleatum* induces chemoresistance through various mechanisms, which we will outline in a systematic manner below.

### 5.1. Inhibition of Apoptosis

*Fusobacterium nucleatum* releases ADP-H into the tumor microenvironment, triggering the ALPK1 (alpha kinase 1) signaling pathway, which activates TIFA (TRAF-interacting protein with FHA domain) and induces strong NF-κB activation [82]. This activation of the TLR4/NF-κB pathway leads to the upregulation of TNFAIP3 and Baculoviral IAP Repeat Containing 3 (BIRC3), a member of the Inhibitor of Apoptosis Proteins (IAP) family [83]. These proteins block caspase-mediated apoptotic processes, enabling colorectal cancer (CRC) cells to avoid cell death and decreasing their responsiveness to chemotherapeutic agents, especially 5-fluorouracil (5-FU) [84] (Table 2).

### 5.2. Promotion of Autophagy

*Fusobacterium nucleatum* promotes chemoresistance in colorectal cancer (CRC) by upregulating autophagy-related proteins such as LC3-II, ULK1, and ATG7, enabling cancer cells to adapt to stress and evade chemotherapy-induced apoptosis. Additionally, it suppresses miRNAs like miR-18a and miR-4802, which typically inhibit autophagy-related genes [41]. This regulation is mediated through TLR4 and MyD88 signaling pathways, further enhancing chemoresistance. Studies indicate that CRC cells exposed to *F. nucleatum* show elevated autophagy indicators (LC3-II, Beclin1) and the metastasis marker Vimentin, with decreased levels of E-cadherin and P62. These changes are mitigated through chloroquine treatment, CARD3 knockdown, or their combination [85,86,87]. Understanding the critical function of autophagy in *F. nucleatum*-associated CRC progression, a new cationic polymer (PNHCQ) has been formulated to inhibit autophagy while delivering plasmid DNA (pDNA) coding for soluble FMS-like tyrosine kinase-1 (sFlt-1), aiming to improve anti-angiogenic treatment [87]. This dual-action system suppresses autophagy while inducing sFlt-1-mediated anti-angiogenic effects, significantly improving therapeutic outcomes in *F. nucleatum*-associated CRC models [87] (Table 2).

### 5.3. Regulation of Anoctamin-1 (ANO1)

*F. nucleatum* has been implicated in the onset and advancement of colorectal cancer (CRC), with studies suggesting its involvement in modulating Anoctamin-1 (ANO1) expression [88]. ANO1, a calcium-activated chloride channel (CaCC), plays a role in key physiological functions and is frequently overexpressed in various cancers, including CRC. Its increased activity is linked to enhanced chloride ion transport, which supports cell proliferation and migration, critical for tumor growth and metastasis [89]. Furthermore, ANO1’s role in regulating cell volume, ion flux, and membrane potential may influence how cells respond to inflammatory environments, potentially aiding cancer survival and progression [90]. Although the precise mechanisms remain unclear, the connection between *Fusobacterium* and ANO1 underscores its importance in CRC pathogenesis [89,91] (Table 2).

### 5.4. Other Mechanisms

*F. nucleatum* plays a significant role in colorectal cancer (CRC) by driving the expansion of cancer stem cells (CSCs) through the upregulation of stemness markers like CD44 and CD133 [4,88,92]. It reshapes lipid metabolism, enhancing fatty acid oxidation in CSCs and boosting fatty acid synthesis in other cancer cells, which strengthens self-renewal and fosters resistance to chemotherapy [92]. Moreover, *F. nucleatum* influences the sonic hedgehog pathway, a critical mechanism for stem cell maintenance, via the MYC/miR-361-5p axis, further enabling the persistence and proliferation of CRC cells under treatment stress [4,93,94,95] (Table 2).

**Table 2:** Molecular mechanisms and results of *Fusobacterium nucleatum* in cancer progression and chemoresistance.

Author, Year	Molecular Mechanisms Analyzed	Results
Martin-Gallausiaux et al., 2024 [82]	Activation of ALPK1/TIFA/NF-κB signaling pathway by *F. nucleatum* through ADP-heptose release.	Increased expression of IL-8, BIRC3, and TNFAIP3; reduced sensitivity to 5-FU; enhanced CRC cell survival and inflammatory responses.
Zhang et al., 2022 [83]	Induction of ALPK1/NF-κB/ICAM1 axis by *F. nucleatum* to enhance CRC cell adhesion and metastasis.	Promoted adhesion of CRC cells to endothelial cells, facilitated metastasis, and correlated high ICAM1 and ALPK1 expression with shorter CRC patient survival.
Zhang et al., 2019 [84]	Modulation of BIRC3 expression via TLR4/NF-κB by *F. nucleatum* to induce chemoresistance to 5-FU in CRC.	High BIRC3 expression reduced CRC cell sensitivity to 5-FU. High *F. nucleatum* abundance correlated with chemoresistance in CRC patients undergoing 5-FU treatment.
Chen Y et al., 2020 [85]	Regulation of CRC metastasis through *F. nucleatum*-mediated CARD3 activation and autophagy pathways.	*F. nucleatum* increased CRC cell motility and metastasis via CARD3, LC3-II, and Beclin1 upregulation; CARD3 knockdown or chloroquine treatment reduced tumor burden and metastasis.
Liu Y et al., 2021 [86]	Induction of chemoresistance in ESCC by *F. nucleatum* through autophagy modulation via ATG7.	*F. nucleatum* promoted chemoresistance to 5-FU, CDDP, and Docetaxel. ATG7 knockdown reversed these effects.
Yang Y et al., 2016 [41]	Upregulation of miR21 via TLR4/MYD88/NF-κB signaling by *F. nucleatum*, leading to CRC progression and invasion.	Increased miR21 expression enhanced proliferation and invasion of CRC cells. High *F. nucleatum* and miR21 levels correlated with reduced RASA1 expression and poor patient outcomes.
Guo S et al., 2022 [90]	Role of ANO1/TMEM16A, a calcium-activated chloride channel, in apoptosis resistance and tumor immune escape.	ANO1 overexpression is driven by 11q13 amplification and influences tumor proliferation, invasion, apoptosis resistance, and immune escape. ANO1 also regulates tumor cell-specific pathways, making it a promising biomarker and therapeutic target.
Lu P. et al., 2019 [89]	Interaction between *F. nucleatum* and ANO1 in promoting chemoresistance in CRC cells.	*F. nucleatum* increased ANO1 expression, reducing apoptosis in CRC cells treated with oxaliplatin and 5-FU. ANO1 knockdown mitigated chemoresistance effects induced by *F. nucleatum*, enhancing chemotherapy-induced apoptosis.
Zhang S et al., 2020 [88]	Induction of epithelial-mesenchymal transition (EMT) by *F. nucleatum* through lncRNA MIR4435-2HG/miR-296-5p/Akt2/SNAI1 signaling in OSCC.	*F. nucleatum* infection promoted cell migration and EMT, with upregulation of mesenchymal markers (N-cadherin, Vimentin, SNAI1) and downregulation of E-cadherin. The MIR4435-2HG/miR-296-5p/Akt2/SNAI1 pathway was implicated in EMT induction, linking *F. nucleatum* infection to oral cancer initiation.
Yu MR, 2020 [92]	Activation of EGFR signaling pathway (AKT, ERK) and promotion of epithelial-mesenchymal transition (EMT).	*Fusobacterium nucleatum* enhances CRC aggressiveness and EMT in DSS-treated cells. In mouse models, *F. nucleatum* increases malignancy in AOM/DSS-induced colon cancer. EGFR inhibition reduces *F. nucleatum*-induced EMT alteration. *F. nucleatum* accelerates CAC progression by activating the EGFR signaling pathway.

## 6. Influence on Immunotherapy Response

In addition to affecting chemotherapy response, *F. nucleatum* also plays a role in modulating the response to immunotherapy [2]. *F. nucleatum* promotes an immunosuppressive milieu by interacting with both tumor cells and immune cells, hindering the effectiveness of immunotherapies such as checkpoint inhibitors (e.g., PD-1/PD-L1 inhibitors), CAR T-cell therapies, and other immune modulators [96,97,98,99,100]. As mentioned earlier, *F. nucleatum*’s role in immune modulation is multifaceted. It enhances the production of pro-inflammatory cytokines and immune-suppressive factors like TGF-β, IL-10, and indoleamine 2,3-dioxygenase (IDO), which collectively foster an immune-tolerant environment [1,4]. This creates challenges for effective immune surveillance, as CRC cells are able to evade recognition by cytotoxic T cells. Additionally, *F. nucleatum* skews the function of tumor-infiltrating lymphocytes (TILs) and promotes the expansion of myeloid-derived suppressor cells (MDSCs) and regulatory T cells (Tregs), both of which dampen anti-tumor immunity [1,101]. Moreover, *F. nucleatum* has been shown to upregulate PD-L1 expression on CRC cells, further enabling tumor cells to evade immune detection and reducing the effectiveness of PD-1/PD-L1-based therapies [96,97,98,99]. In Table 3, the most recent studies on the influence of *Fusobacterium nucleatum* on immunotherapy in CRC are listed.

## 7. Conclusions

In conclusion, the growing body of research has shed light on the complex molecular mechanisms by which *F. nucleatum* contributes to CRC progression and chemoresistance. *F. nucleatum* not only enhances CSC stemness and promotes aggressive tumor behavior, but also plays a critical role in mediating resistance to common chemotherapies such as oxaliplatin and 5-FU [102]. Emerging therapeutic strategies that target *F. nucleatum* and its associated pathways hold great promise in overcoming these challenges. Pharmacological interventions like metformin, which suppress *F. nucleatum*-induced stemness and enhance chemosensitivity, and Br-J-I, which exhibits antimicrobial activity against *F. nucleatum* and synergizes with 5-FU, demonstrate early potential for improving treatment outcomes [95]. Additionally, innovative drug delivery systems, including tumor-targeted nanoassemblies and phage-guided hybrid nanomaterials, have shown efficacy in selectively targeting and eliminating *F. nucleatum*, thereby improving the effectiveness of chemotherapy [103].

In the realm of immunotherapy, *F. nucleatum*-directed vaccination strategies and microbial ecosystem replacement offer intriguing possibilities for modulating the tumor microbiome, although further research is needed to address the challenges posed by *F. nucleatum*’s ability to evade immune responses [4,104,105]. Targeting key proteins and pathways, such as Annexin A1 and BIRC3, which are upregulated in *F. nucleatum*-infected CRC cells, provides additional therapeutic avenues to overcome *F. nucleatum*-mediated chemoresistance [106,107]. Moreover, miRNA-based therapeutics, which influence *F. nucleatum* proliferation and resistance, could further complement current treatment strategies [107,108,109].

Future advancements in designing personalized treatments for CRC patients with elevated *F. nucleatum* levels are key to enhancing therapeutic success. Integrating standard chemotherapies with therapies targeting *F. nucleatum* may overcome resistance mechanisms that currently hinder their efficacy. Detailed investigations into the complex relationships among *F. nucleatum*, CSCs, and the tumor microenvironment are critical. Clinical trials assessing *F. nucleatum*-focused interventions, such as microbiota therapies, vaccines, and cutting-edge nanomaterials, will be vital in transforming these encouraging preclinical insights into effective clinical treatments. Such efforts hold the potential to address the therapeutic obstacles posed by *F. nucleatum* in CRC and improve outcomes for patients facing this challenging disease.

## Figures and Tables

**Table 1:** The impact of *Fusobacterium nucleatum* in recruitment and modulation of immunosuppressive cells.

Cells	Mechanism	References
MDSCs	*F. nucleatum* selectively recruits myeloid-derived suppressor cells (MDSCs) to the TME.	[1,50]
T cell proliferation	MDSCs inhibit T cell proliferation and induce apoptosis through iNOS and arginase-1.	[51,52]
TAMs, neutrophils, regulatory DCs	*F. nucleatum* recruits tumor-associated macrophages (TAMs), neutrophils, and regulatory DCs that promote inflammation, angiogenesis, invasion, and metastasis.	[53,54]
Tumor-associated neutrophils (TANs)	*F. nucleatum* drives tumor-associated neutrophils (TANs) toward a pro-tumor N2 phenotype via TGF-β signaling.	[55,56]
N2 TANs	N2 TANs exacerbate tumorigenesis by producing reactive oxygen species (ROS), which cause DNA damage and enhance tumor progression.	[55,56]
M2 macrophages	Macrophages influenced by *F. nucleatum* via TLR4 signaling shift toward an M2-like phenotype, aiding tumor progression.	[57,58]
CD4+ T cells	*F. nucleatum* reduces the density of CD4+ T cells in tumors compared to normal tissues, suppressing T helper cell-mediated immune responses.	[59,60]

**Table 3:** Molecular mechanisms and results of *Fusobacterium nucleatum*’s role in immunotherapy for CRC.

Author, Year	Aim and Molecular Mechanisms Analyzed	Results
Ding T. et al., 2024 [96]	Investigate resistance to PD-1/PD-L1 blockade in CRC and *F. nucleatum*’s role.	*F. nucleatum* infection increased sensitivity to PD-L1 blockade via immune cell accumulation. Targeting *F. nucleatum* may overcome resistance.
Wang X. et al., 2024 [97]	Explore how *Fusobacterium nucleatum* sensitizes MSS CRC to anti-PD-1 therapy.	*F. nucleatum* produces butyric acid, inhibiting HDAC3/8 in CD8+ T cells, enhancing effector functions and alleviating exhaustion. High intratumoral *F. nucleatum* predicts better therapy response.
Ugai T. et al., 2023 [98]	Assess the relationship between tumor CD274 expression and *F. nucleatum* abundance in CRC.	Tumor CD274 expression was inversely associated with *F. nucleatum* levels, suggesting distinct immunosuppressive strategies in tumor subgroups.
Jang S.S. et al., 2023 [100]	Investigate how *F. nucleatum* and succinic acid influence resistance to anti-PD-1 therapy in CRC.	*F. nucleatum*-derived succinic acid suppresses cGAS-interferon-β pathway, reducing CD8+ T cell trafficking to the TME. Antibiotic treatment resensitizes tumors to immunotherapy.
Gao Y. et al., 2021 [99]	Investigate the effect of *F. nucleatum* on PD-L1 therapy in CRC.	High *F. nucleatum* levels correlated with better response to PD-1 blockade, enhancing antitumor effects through STING signaling and increased IFN-γ+ CD8+ TILs.
